# Supplementary figures and images for: Priority effects, nutrition and milk glycan-metabolic potential drive Bifidobacterium longum subspecies dynamics in the infant gut microbiome
Source: PeerJ. 2025 Jan 21;13:e18602. doi: 10.7717/peerj.18602 (PMC11758915; doi:10.7717/peerj.18602)

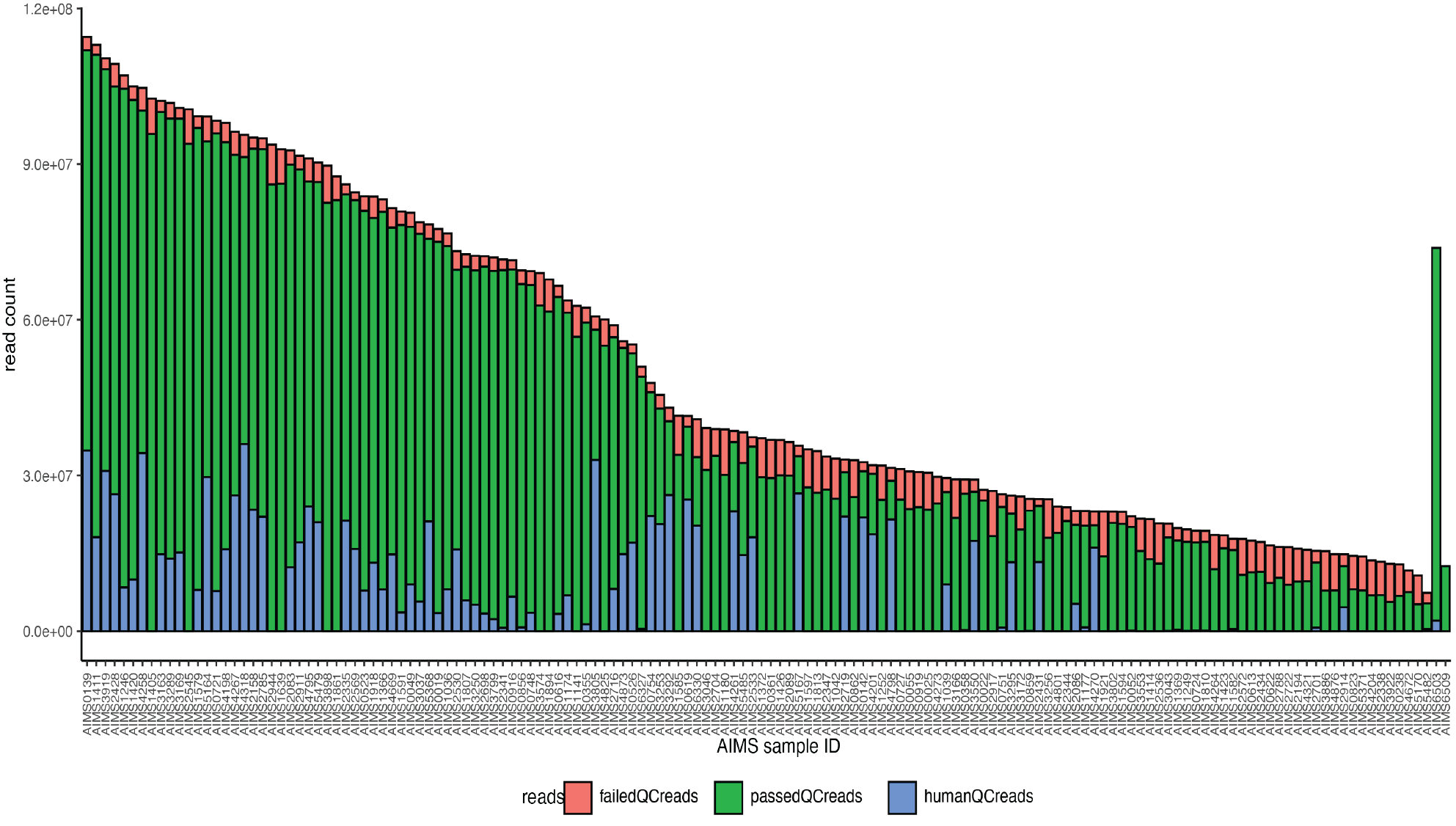

Supplement: Supplemental Information 1 — Overview of the number of reads retained per sample. For each bar, the colors indicate the proportion of reads that passed (green) and failed (red) quality control as well as reads mapping to human DNA (blue). Failed and human reads were subsequently filtered out the dataset. [file peerj-13-18602-s001.png]

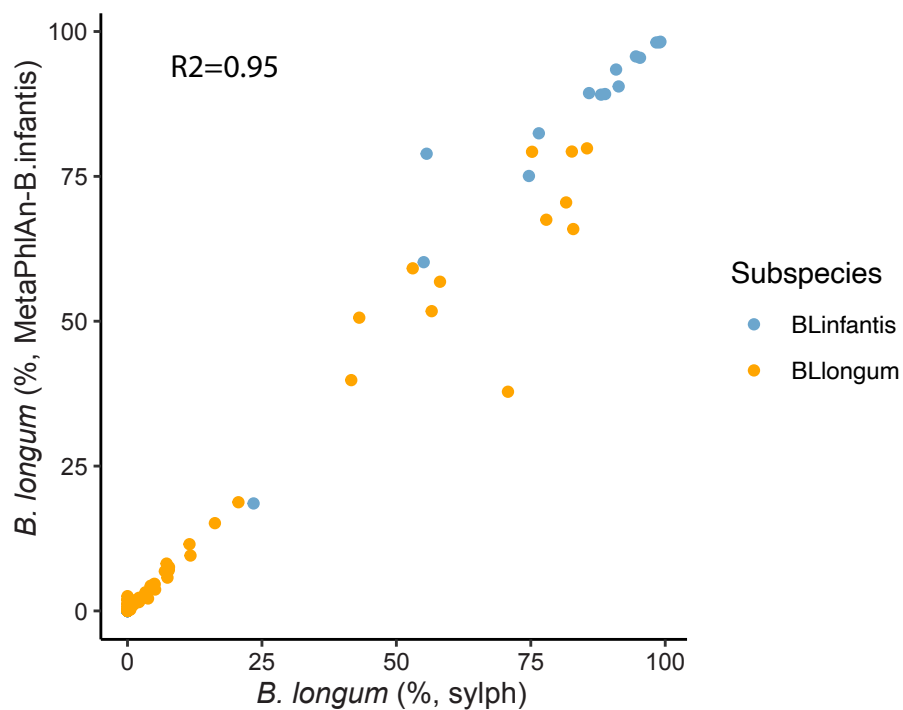

Supplement: Supplemental Information 5 — The correlation dot plot shows the relative abundances of BLlongum (orange) and BLinfantis (light blue) detected in AIMS infants and estimated using sylph v.0.6.1 and MetaPhlAn v4.0.6 with the amended MetaPhlAn-B.infantis database. [file peerj-13-18602-s005.pdf]

A

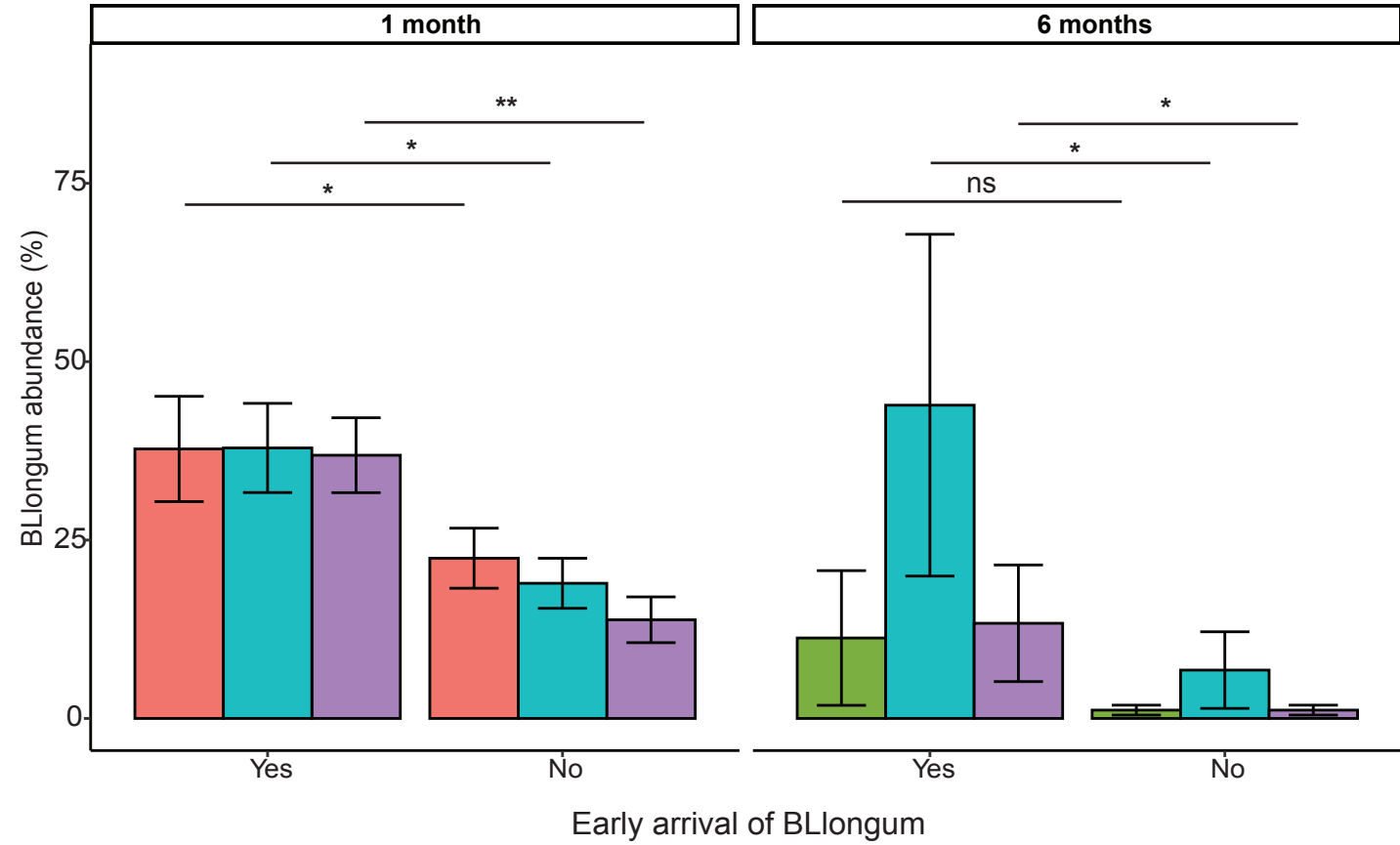

B

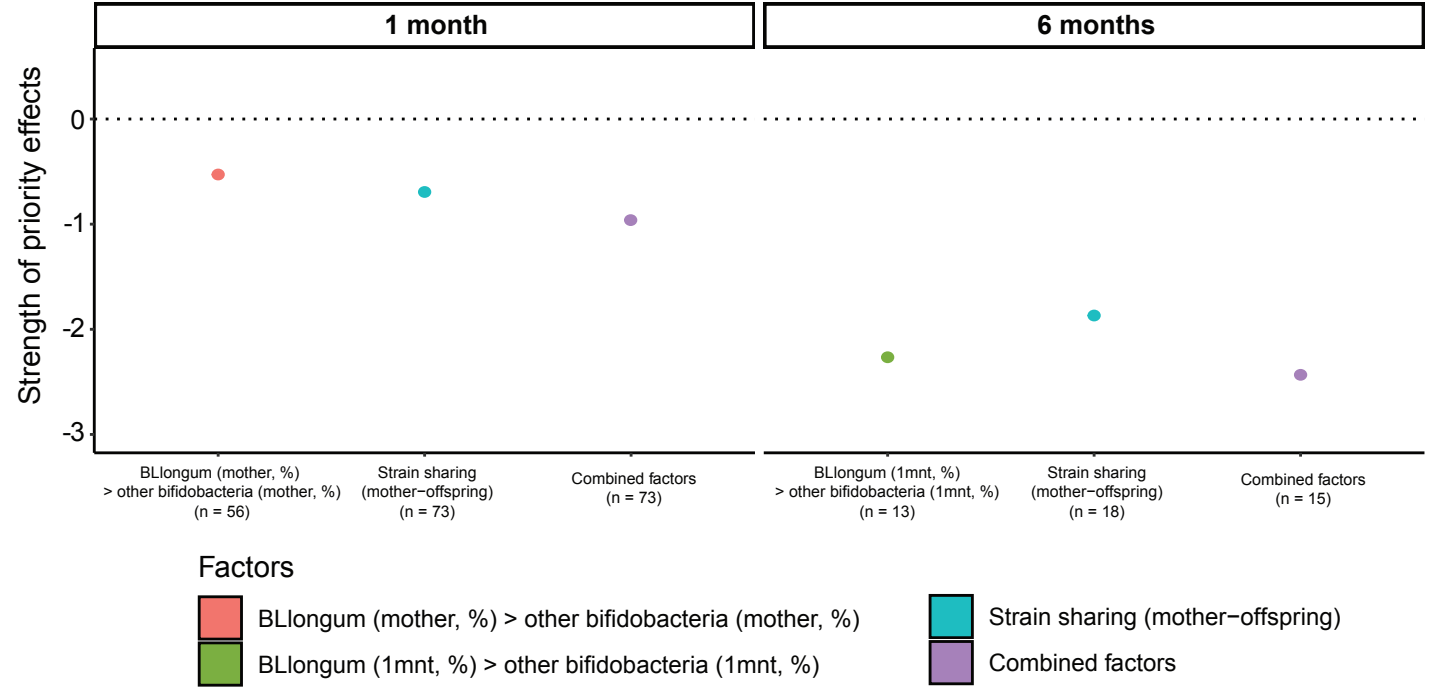

Supplement: Supplemental Information 15 — (A) Bargraphs summarize the relative abundances of BLlongum in the gut of one and six-month-old infants from AIMS and publicly available datasets used in this study, based on i) ‘Mother-Infant Strain Sharing’, ii) ‘BLlongum (mother or 1 month, %) ¿other bifidobacteria (mother or 1 month, %)’ and iii) if either i) or ii) are true. Significance between groups was calculated using Wilcoxon’s signed-rank tests: * p < 0.05, ** p < 0.01, ns = not significant. (B) Strength of priority effect quantified using the method by Vannette & Fukami (2014) and estimated for maternal factors identified in this study as proxies for priority effects. Negative values indicate that priority effects are in place for a specific proxy. [file peerj-13-18602-s015.pdf]
