# Supplementary material for: Priority effects, nutrition and milk glycan-metabolic potential drive Bifidobacterium longum subspecies dynamics in the infant gut microbiome
Source: PeerJ. 2025 Jan 21;13:e18602. doi: 10.7717/peerj.18602 (PMC11758915; doi:10.7717/peerj.18602)
Supplement: Supplemental Information 3 — Phylogenomics tree based on concatenated alignment of 172 single-copy core genes from 63 B. longum MAGs, four B. longum reference genomes (BLlongum = 2, BLinfantis = 2) and one B. adolescentis genome (outer group) used in this study. Differentiation of B. longum subspecies was based on phylogenomics clustering, where MAGsclustering with a specific reference genome were subsequently assigned to the respective subspecies. [file peerj-13-18602-s003.pdf]

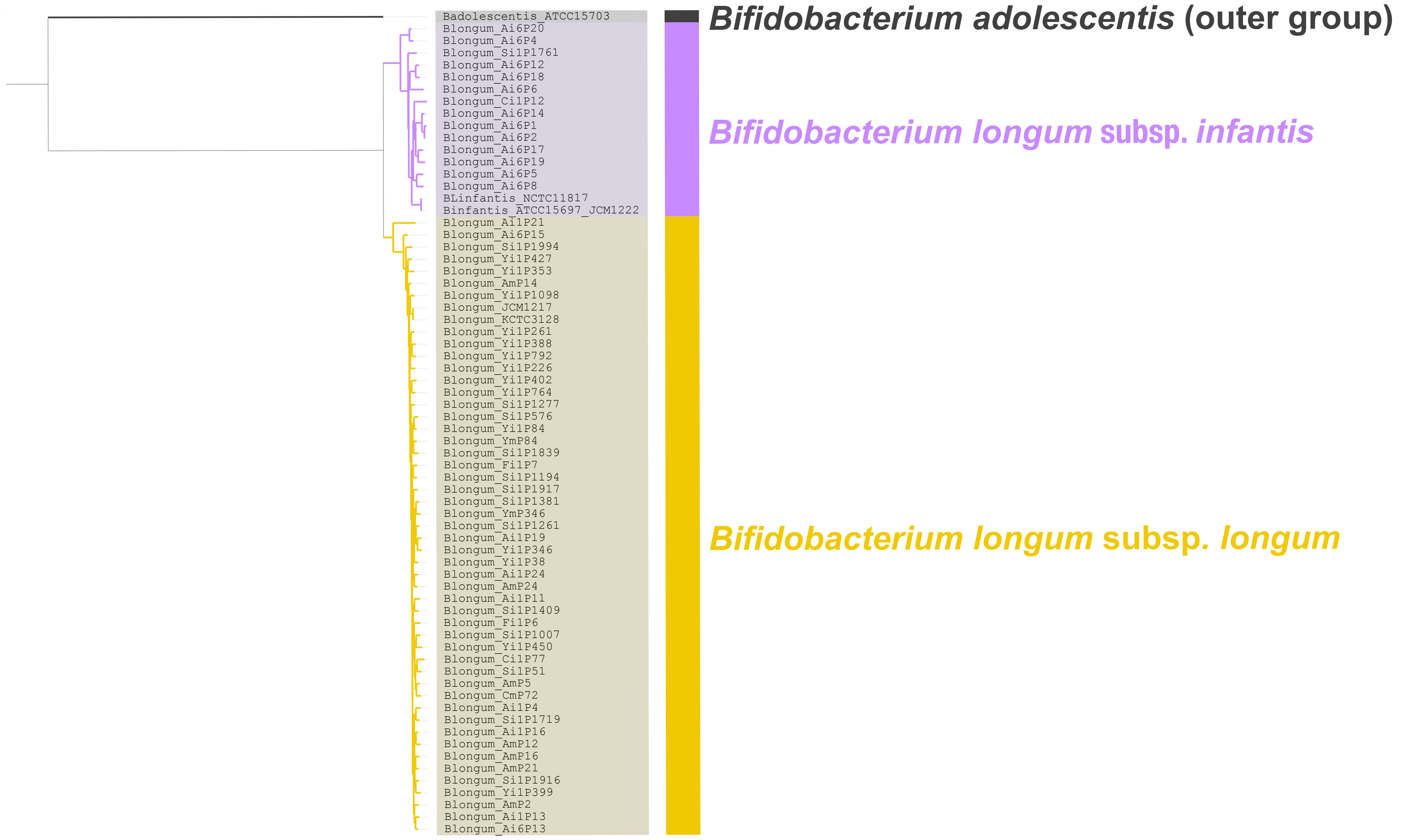

Badolescentis_ATCC15703
Blongum_Ai6P20
Blongum_Ai6P4
Blongum_Si1P1761
Blongum_Ai6P12
Blongum_Ai6P18
Blongum_Ai6P6
Blongum_Ci1P12
Blongum_Ai6P14
Blongum_Ai6P1
Blongum_Ai6P2
Blongum_Ai6P17
Blongum_Ai6P19
Blongum_Ai6P5
Blongum_Ai6P8
BLinfantis_NCTC11817
Binfantis_ATCC15697_JCM1222
Blongum_Ai1P21
Blongum_Ai6P15
Blongum_Si1P1994
Blongum_Yi1P427
Blongum_Yi1P353
Blongum_AmP14
Blongum_Yi1P1098
Blongum_JCM1217
Blongum_KCTC3128
Blongum_Yi1P261
Blongum_Yi1P388
Blongum_Yi1P792
Blongum_Yi1P226
Blongum_Yi1P402
Blongum_Yi1P764
Blongum_Si1P1277
Blongum_Si1P576
Blongum_Yi1P84
Blongum_YmP84
Blongum_Si1P1839
Blongum_Fi1P7
Blongum_Si1P1194
Blongum_Si1P1917
Blongum_Si1P1381
Blongum_YmP346
Blongum_Si1P1261
Blongum_Ai1P19
Blongum_Yi1P346
Blongum_Yi1P38
Blongum_Ai1P24
Blongum_AmP24
Blongum_Ai1P11
Blongum_Si1P1409
Blongum_Fi1P6
Blongum_Si1P1007
Blongum_Yi1P450
Blongum_Ci1P77
Blongum_Si1P51
Blongum_AmP5
Blongum_CmP72
Blongum_Ai1P4
Blongum_Si1P1719
Blongum_Ai1P16
Blongum_AmP12
Blongum_AmP16
Blongum_AmP21
Blongum_Si1P1916
Blongum_Yi1P399
Blongum_AmP2
Blongum_Ai1P13
Blongum_Ai6P13
Bifidobacterium adolescentis (outer group)
Bifidobacterium longum subsp. *infantis*
Bifidobacterium longum subsp. *longum*
