# Supplementary material for: Priority effects, nutrition and milk glycan-metabolic potential drive Bifidobacterium longum subspecies dynamics in the infant gut microbiome
Source: PeerJ. 2025 Jan 21;13:e18602. doi: 10.7717/peerj.18602 (PMC11758915; doi:10.7717/peerj.18602)
Supplement: Supplemental Information 4 — The heatmap shows the distribution of B. longum subspecies-specific marker genes (119 BLinfantis and 128 BLlongum; MetaPhlAn-B.infantis database) among 63 MAGs from AIMS and publicly available studies. Clustering of MAGs based on the copy-number of marker genes was used to differentiate BLlongum and BLinfantis MAGs and validate subspecies assignment achieved through our phylogenomics method. [file peerj-13-18602-s004.pdf]

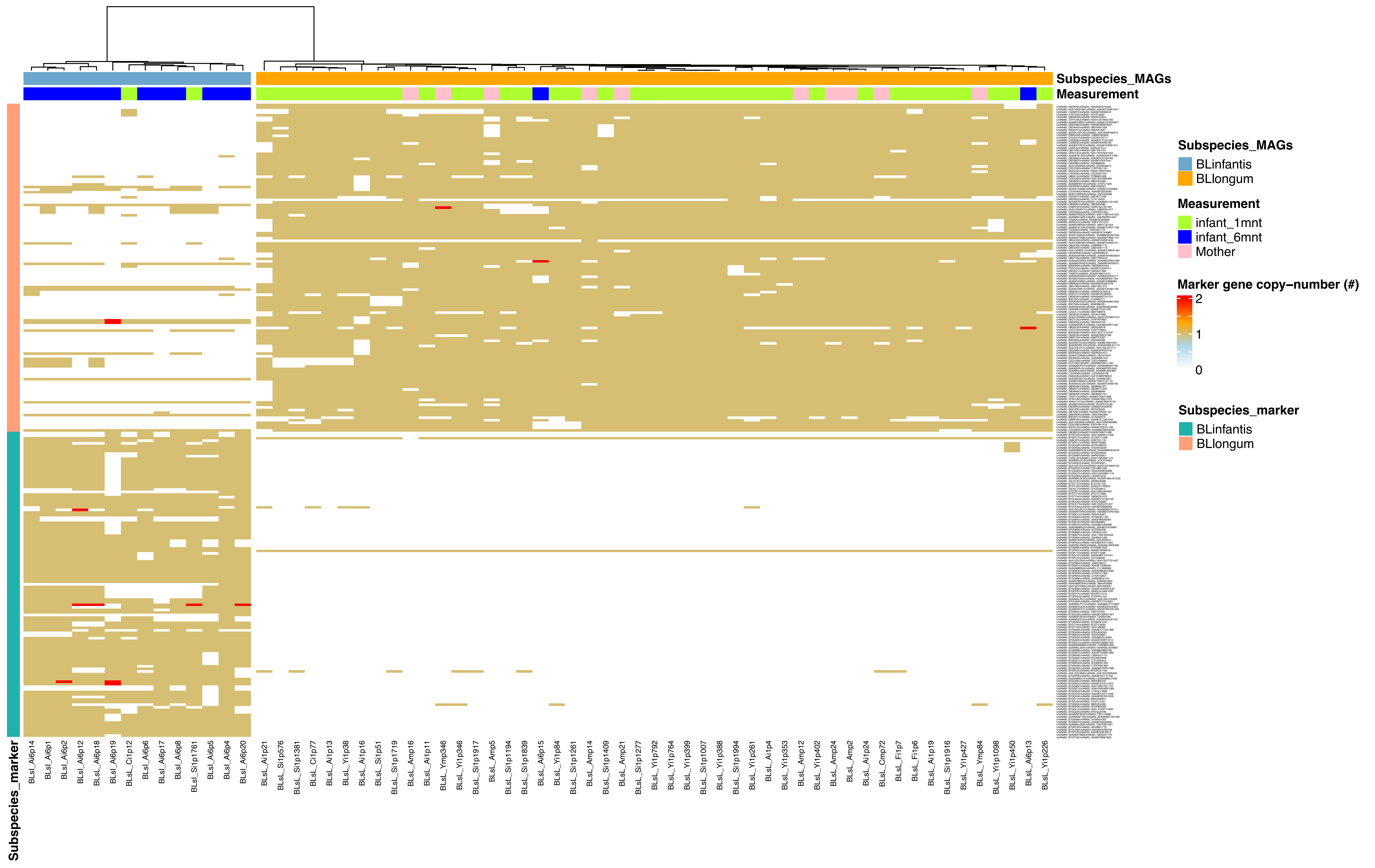

Subspecies_MAGs
Measurement
Subspecies_marker
Subspecies_MAGs
BLinfantis
BLlongum
Measurement
infant_1mnt
infant_6mnt
Mother
Marker gene copy−number (#)
2
1
0
Subspecies_marker
BLinfantis
BLlongum
BLsl_Ai6p14
BLsl_Ai6p1
BLsl_Ai6p2
BLsl_Ai6p12
BLsl_Ai6p18
BLsl_Ai6p19
BLsl_Ci1p12
BLsl_Ai6p6
BLsl_Ai6p17
BLsl_Ai6p8
BLsl_Si1p1761
BLsl_Ai6p5
BLsl_Ai6p4
BLsl_Ai6p20
BLsl_Ai1p21
BLsl_Si1p576
BLsl_Si1p1381
BLsl_Ci1p77
BLsl_Ai1p13
BLsl_Yi1p38
BLsl_Ai1p16
BLsl_Si1p51
BLsl_Si1p1719
BLsl_Amp16
BLsl_Ai1p11
BLsl_Ymp346
BLsl_Yi1p346
BLsl_Si1p1917
BLsl_Amp5
BLsl_Si1p1194
BLsl_Si1p1839
BLsl_Ai6p15
BLsl_Yi1p84
BLsl_Si1p1261
BLsl_Amp14
BLsl_Si1p1409
BLsl_Amp21
BLsl_Si1p1277
BLsl_Yi1p792
BLsl_Yi1p764
BLsl_Yi1p399
BLsl_Si1p1007
BLsl_Yi1p388
BLsl_Si1p1994
BLsl_Yi1p261
BLsl_Ai1p4
BLsl_Yi1p353
BLsl_Amp12
BLsl_Yi1p402
BLsl_Amp24
BLsl_Amp2
BLsl_Ai1p24
BLsl_Cmp72
BLsl_Fi1p7
BLsl_Fi1p6
BLsl_Ai1p19
BLsl_Si1p1916
BLsl_Yi1p427
BLsl_Ymp84
BLsl_Yi1p1098
BLsl_Yi1p450
BLsl_Ai6p13
BLsl_Yi1p226
