# Supplementary material for: Priority effects, nutrition and milk glycan-metabolic potential drive Bifidobacterium longum subspecies dynamics in the infant gut microbiome
Source: PeerJ. 2025 Jan 21;13:e18602. doi: 10.7717/peerj.18602 (PMC11758915; doi:10.7717/peerj.18602)
Supplement: Supplemental Information 2 — Each layer represents a B. longum MAG and the dark coloration within each layer indicates the abundance of a gene cluster (GC). The subsequent 8 layers correspond to various statistics related to the analysis i.e. the number of contributing genomes per GC, number of genes per GC and different levels of KEGG and COG functional annotations. Additionally, we report MAG’s total length, completeness, redundancy, GC total number and content and HMG-profile affiliation: BLinfantis-HMG (purple, number of genomes=14), BLlongum-HMGsimple (green,n=24), BLlongum-HMGcomplex (orange, n=25), reference genomes (red, n=4). The dendrogram above the layers indicates the similarities among genomes based on GC presence/absence. Two major clusters are visible: a BLinfantis (purple) and BLlongum (dark yellow) cluster. [file peerj-13-18602-s002.pdf]

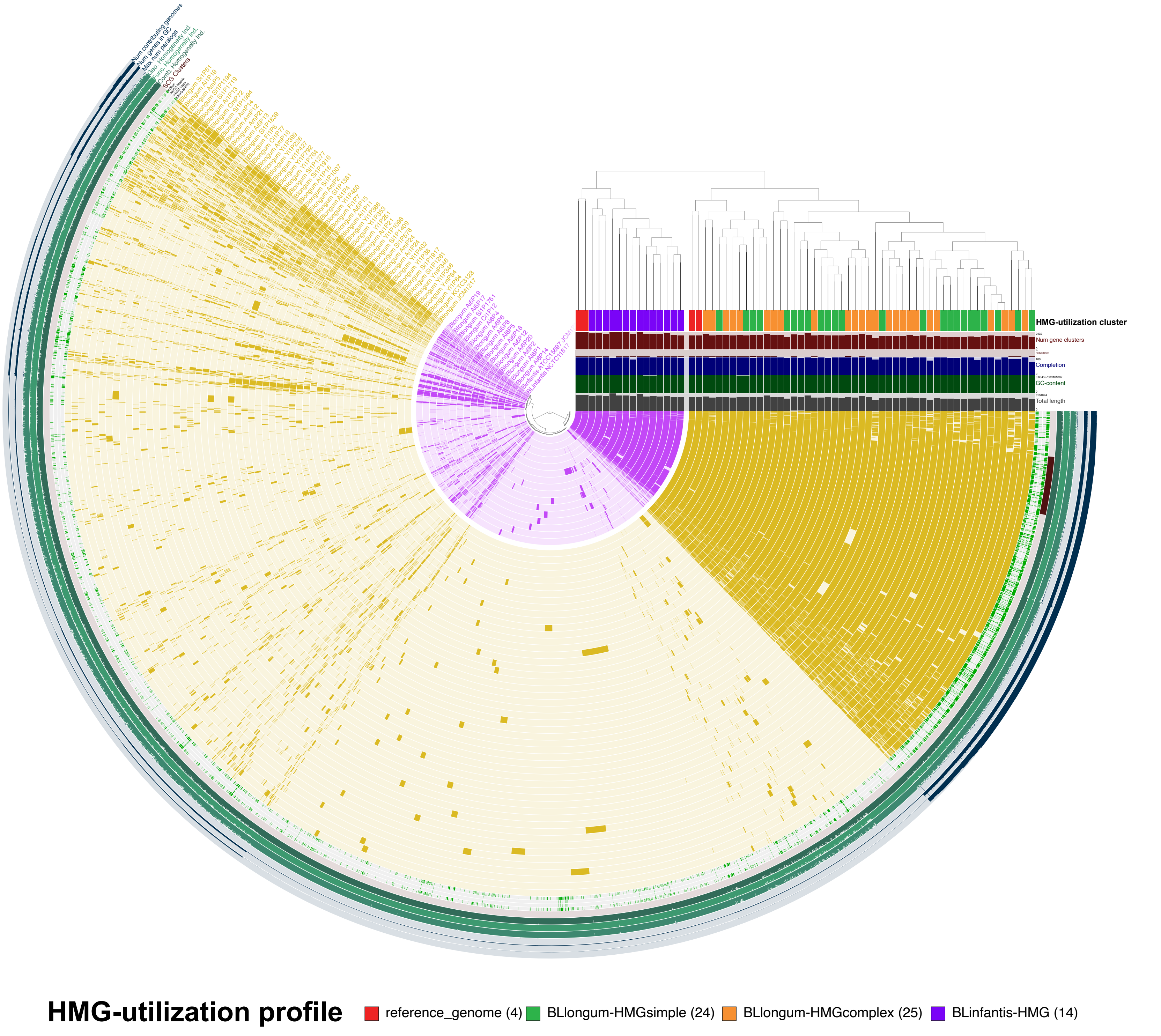
HMG-utilization cluster
Num gene clusters
Completion
GC-content
Total length
HMG-utilization profile
reference_genome (4)
BLlongum-HMGsimple (24)
BLlongum-HMGcomplex (25)
BLinfantis-HMG (14)
